# Solar Prominence Modelling and Plasma Diagnostics at ALMA Wavelengths

**DOI:** 10.1007/s11207-017-1161-9

**Published:** 2017-09-04

**Authors:** Andrew Rodger, Nicolas Labrosse

**Affiliations:** SUPA, School of Physics & Astronomy, University of Glasgow, Glasgow, G12 8QQ Scotland UK

**Keywords:** Corona, radio emission, Prominences, models, Radio emission, quiet, Spectrum, continuum

## Abstract

Our aim is to test potential solar prominence plasma diagnostics as obtained with the new solar capability of the *Atacama Large Millimeter/submillimeter Array* (ALMA). We investigate the thermal and plasma diagnostic potential of ALMA for solar prominences through the computation of brightness temperatures at ALMA wavelengths. The brightness temperature, for a chosen line of sight, is calculated using the densities of electrons, hydrogen, and helium obtained from a radiative transfer code under non-local thermodynamic equilibrium (non-LTE) conditions, as well as the input internal parameters of the prominence model in consideration. Two distinct sets of prominence models were used: isothermal-isobaric fine-structure threads, and large-scale structures with radially increasing temperature distributions representing the prominence-to-corona transition region. We compute brightness temperatures over the range of wavelengths in which ALMA is capable of observing (0.32 – 9.6 mm), however, we particularly focus on the bands available to solar observers in ALMA cycles 4 and 5, namely 2.6 – 3.6 mm (Band 3) and 1.1 – 1.4 mm (Band 6). We show how the computed brightness temperatures and optical thicknesses in our models vary with the plasma parameters (temperature and pressure) and the wavelength of observation. We then study how ALMA observables such as the ratio of brightness temperatures at two frequencies can be used to estimate the optical thickness and the emission measure for isothermal and non-isothermal prominences. From this study we conclude that for both sets of models, ALMA presents a strong thermal diagnostic capability, provided that the interpretation of observations is supported by the use of non-LTE simulation results.

## Introduction

The temperature structure of solar prominences remains an important question in solar physics. Prominences are cool, dense structures suspended in the hot sparse corona, and it is generally accepted that there is a prominence-to-corona transition region (PCTR) between the two regimes. The importance of the PCTR in prominence modelling has been discussed by Anzer and Heinzel (1999), and its effect on various spectral lines demonstrated by *e.g.* Heinzel *et al.* (2001), Labrosse and Gouttebroze (2004), Labrosse *et al.* (2010), Heinzel *et al.* (2015b). However, the nature of the PCTR and its relationship to the prominence and prominence fine-structure is not fully understood. To address this, an accurate and reliable temperature diagnostic, capable of resolving fine-structure, is required.

Numerous studies of solar prominences have allowed the physical parameters of the prominence plasma to be evaluated through analysing the shape and intensity of spectral lines (Labrosse *et al.*, 2010; Parenti, 2014; Labrosse, 2015). However, the complex mechanisms involved in the spectral line and continuum formation at optical or UV wavelengths in prominences presents several issues when attempting to accurately measure any kinetic temperature distribution. Optically thick radiation requires a complex non-LTE radiative transfer treatment, which makes temperature estimations problematic. In addition, optically thick line profiles are susceptible to broadening effects, *e.g.* they become non-Gaussian, masking the true thermally broadened profile. As for optically thin radiation, any discernible temperature structure results from the cumulative effect from across the entire line of sight (LOS), thus information on any fine-structures present is lost. In reality, observations in optically thick lines will be affected by a mixture of these effects, as one integrates through more and more fine structures when observing in the optically thin line wings (Gunár *et al.*, 2008; Labrosse and Rodger, 2016).

In radio wavelengths, temperature measurements are considerably more simple (*e.g.* Loukitcheva *et al.*, 2004; Heinzel *et al.*, 2015a; Wedemeyer *et al.*, 2016). In the solar millimeter/submillimeter (mm/sub-mm) domain the dominant emission mechanism is free-free collisional processes. The source function hence results from local thermodynamic equilibrium (LTE) conditions and is thus Planckian. In the Rayleigh–Jeans domain this means that the source function varies linearly with kinetic temperature. This causes the peak contribution function of the continuum radiation to be highly dependent on the local temperature, leading to the often used term “linear thermometer”. Loukitcheva *et al.* (2015) concluded that for chromospheric radiation, brightness temperatures at mm wavelengths provide a reasonable measure of the thermal structure, up to resolutions of $1''$. In the context of solar flare models, Heinzel and Avrett (2012) synthesised the thermal continua from the optical to the mm radio, demonstrating how these continua are formed, and again showing the close correspondence between brightness temperature and kinetic temperature.

Despite the clear advantages presented with measurements at mm radio wavelengths, observations of solar prominences have so far been limited by low spatial resolutions (*e.g.* Vrsnak *et al.*, 1992; Bastian, Ewell, and Zirin, 1993; Harrison *et al.*, 1993; Irimajiri *et al.*, 1995). Studies of prominences and filaments at centimeter wavelengths have also been conducted (see *e.g.* Chiuderi Drago *et al.*, 2001). Now, with the use of the *Atacama Large Millimeter/Submillimeter Array* (Karlický *et al.*, 2011, ALMA), solar observations with unprecedentedly high spatial resolution may be obtained. A review of ALMA’s potential contribution to solar physics is presented in Wedemeyer *et al.* (2016).

ALMA will offer the opportunity of a new approach to the observation and study of solar prominences. It is thus important to understand how we expect prominences and prominence fine structure to appear in brightness temperature when observed in ALMA’s wavelength range. Once prominence observations are obtained, it will also be important to understand how to use such measurements to infer information about the temperature and plasma structures in question. The visibility of prominences beyond the limb in both ALMA Bands 3 and 6 has recently been indicated by recent ALMA science verification full-disk imaging results (White *et al.*, 2017; Alissandrakis *et al.*, 2017).

A study of how prominences may appear as viewed through ALMA was conducted by Heinzel *et al.* (2015a). This was done by taking an $\mbox{H}\upalpha$ coronagraph image, and using the empirical relation between $\mbox{H}\upalpha$ intensity and emission measure, estimating the brightness temperature for such a plasma. These brightness temperatures were tested using the *Common Astronomy Software Applications* (CASA) package to simulate ALMA observations. Assumptions were required, however, including the use of a simple temperature structure for the prominence, whilst the simulated ALMA observations were restricted by the resolution of the instrument used to create the original $\mbox{H}\upalpha$ observation.

Simulated observations of whole prominences in the mm domain have been created by Gunár *et al.* (2016) using a 3D whole-prominence fine-structure model. The prominence fine structures are formed within dips in a synthetic prominence magnetic field. From the material within the fine structure the hydrogen free-free extinction coefficient and thus the brightness temperature are calculated. This model is used to visualise the brightness temperature and optical thicknesses of prominences on the limb and on-disk filaments at a range of ALMA wavelengths. The authors underline the requirement for mm observations in both optically thin and optically thick wavelengths for observations of filaments in order to distinguish between sparse, low-emitting material and dense, high-absorbing material.

In this study we test the diagnostic potential of ALMA for solar prominences by computing brightness temperatures using the 2D cylindrical solar prominence models of Gouttebroze and Labrosse (2009). We consider two specific sets of prominence models: isothermal and isobaric fine structures, and non-isothermal large-scale structures. These sets of models have been designed to replicate different prominence descriptions. The isothermal-isobaric fine-structure models correspond to individual threads of varying temperature or pressure, whilst the large-scale non-isothermal cases describe a prominence with a cool thread core surrounded by a sheath of increasingly hot PCTR material. For each of these cases, we investigate the temperature and plasma diagnostic capability of the brightness temperature measurements.

In Section 2 we provide a description of the millimeter/sub-millimeter prominence models used in this study. Section 3 presents brightness temperatures for both isothermal-isobaric fine-structure and non-isothermal large-scale prominence models, discussing their use as thermal diagnostics. In Section 4 we investigate the brightness temperature as a diagnostic for plasma properties such as optical thickness and emission measure. Section 5 presents our conclusions from the results of this study and gives a discussion of their implications for solar observations with ALMA.

## Modelling

In this study we have chosen to model solar prominences and their associated fine-structures using 2D cross-sections of cylindrical structures. Cylindrical models have the benefit of being able to consider non-uniform incident radiation on the structure as well as variations in LOS length from core to the PCTR. Such cylindrical structures well represent magnetic flux-tube fine structures as are present in active region prominences. Whole prominences are not cylindrical, and quiescent prominence fine-structures are expected to be composed of plasma that is supported in magnetic dips. However, a 2D cross-section of a cylinder can be used as a suitable approximation. We used the 2D cylindrical radiative transfer code for this, which considers both hydrogen and helium, of Gouttebroze and Labrosse (2009), referred to as C2D2E. It considers a 5-level plus continuum hydrogen atom and a 33-level plus continuum helium atom, including 29 levels for He I and 4 levels for He II. The electron density is calculated through the ionisation equilibrium of a pure hydrogen and helium plasma. Through iteratively solving the radiation transfer and statistical equilibrium equations, the code computes the non-LTE energy level population densities of H and He, the electron density, and the specific intensities of several spectral lines and continua.

### Input Parameters

The input to C2D2E can consider a range of intrinsic thread parameters. Geometric variables include altitude above solar disc, inclination angle, and thread diameter. Internal thread parameters are gas pressure ($P_{\mathrm{g}}$), temperature ($T$), and helium abundance ratio ($A_{\mathrm{He}}$). In this study we consider fine-structure isothermal-isobaric prominence threads, as well as larger-scale threads with radially increasing temperature distributions.

High-resolution observations of solar prominences reveal increasing degrees of fine structure. Some models suggest that prominences may be described as collections (or bundles) of fine-structure threads, either with individually varying temperatures, or with an overlying PCTR region (Fontenla *et al.*, 1996; Gunár *et al.*, 2008; Labrosse and Rodger, 2016). ALMA has the potential, with the correct array configurations, to observe with resolutions of $0.015''\,\mbox{--}\,1.4'' \times\lambda_{\mathrm{mm}}$ (Bastian, 2002), and thus the potential capability to observe such fine-structure threads individually; for example, a 500km wide fine-structure thread would have an angular size of ${\approx}\,0.7''$. To model prominence fine-structure, we have assumed both isothermal temperature and isobaric pressure distributions. This is a valid assumption because over comparatively small distances such as are presented in fine-structure observations, temperature and pressure variations may be small. To investigate isothermal-isobaric fine structures, we have created a model grid with six temperatures by five pressures to analyse. The input parameters are listed in Table 1. The helium abundance and microturbulent velocity are set to 0.1 and $5~\mbox{km}\,\mbox{s}^{-1}$ respectively.

**Table 1:** Parameters for isothermal-isobaric fine-structure models.

Parameter	Value
Temperature (K)	{5000, 6000, 7000, 8000, 9000, 10 000}
Pressure (dyn cm^−2^)	{0.02, 0.05, 0.1, 0.3, 0.5}
Radius (km)	250

In observations of larger or less resolved structures, it might not be prudent to assume an isothermal temperature distribution. For these cases we consider larger scale, full-prominence width threads with distinct core and PCTR regions. The prominence core is defined by an isothermal temperature distribution, whilst the temperature in the PCTR increases with radius. Constant gas pressure is assumed across the cylinder. In all non-isothermal models an *ad-hoc* temperature gradient for the PCTR is considered, with the form

1 $$ \log{T(r)} = \log{T_{0}} + (\log{T_{1}} - \log{T_{0}}) \frac{r-r_{0}}{r_{1}-r_{0}} , $$

where $T_{0}$ and $T_{1}$ are the kinetic temperatures of the thread core and surrounding corona, respectively. The inner radius of the transition region is defined by $r_{0}$, whilst the radius of the cylinder is $r_{1}$. Within the isothermal core ($r < r_{0}$), the temperature of the plasma is fixed at $T = T_{0}$. This temperature distribution is not generated from any theoretical model and simply serves the purpose of showing the effect of a radial temperature gradient. Table 2 gives the parameters used to define this set of models. The helium abundance and microturbulent velocity are again fixed at 0.1 and $5~\mbox{km}\,\mbox{s}^{-1}$, respectively.

**Table 2:** Parameters for large-scale, non-isothermal models.

Parameter	Value
Temperature (K)	$T_{0} = 6 \times 10^{3}$, $T_{1} = 1 \times 10^{5}$
Pressure (dyn cm^−2^)	{0.02, 0.03, 0.05, 0.1, 0.2, 0.3, 0.5}
Inner radius (km)	500
Outer radius (km)	1000

**Figure 1:**
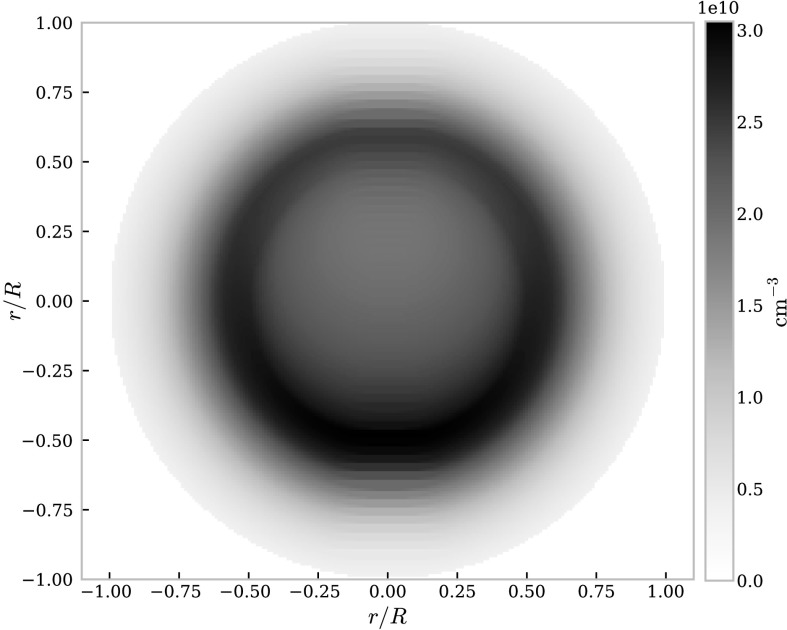
Electron density distribution for a non-isothermal large-scale prominence as described in Table 2 with a pressure of $0.1~\mbox{dyn}\,\mbox{cm}^{-2}$.

A plot showing the variation in electron density across a non-isothermal, large scale prominence thread with a pressure of $0.1~\mbox{dyn}\,\mbox{cm}^{-2}$ is shown in Figure 1. This figure shows the importance of the ionising incident radiation on the lower boundary of the thread on the electron and thus ion densities.

### Millimeter/Sub-millimeter Continuum Formation in Solar Prominences

In computing the radiation at a given wavelength, the most import aspect to consider is the emission mechanisms. ALMA will take observations in the wavelength range between 0.3 mm and 9.0 mm (Karlický *et al.*, 2011). However, initial solar observations in Cycle 4 are limited to 2.6 – 3.6 mm (Band 3) and 1.1 – 1.4 mm (Band 6). At mm/sub-mm wavelengths the Sun’s radiation is dominated by the free-free thermal continuum (Wedemeyer *et al.*, 2016). Free-free processes are fully collisional, hence the continuum radiation is formed under local thermodynamic equilibrium (LTE) conditions. The respective source function is thus given by the Planck function, assuming the velocity distribution is Maxwellian. When we use the Rayleigh–Jeans law, the continuum source function $S_{\nu}$ is therefore

2 $$ S_{\nu} = \frac{2\nu^{2}k_{\mathrm{B}}T}{c^{2}} , $$

where $\nu$ is the frequency, $T$ is the kinetic temperature, and $k_{\mathrm{B}}$ and $c$ are the Boltzmann constant and the speed of light in a vacuum, respectively.

The continuum intensity emitted in LTE over a given optical depth at frequency $\nu$ is described by

3 $$ I_{\nu} = \int S_{\nu} \mathrm{e}^{-\tau_{\nu}}\,\mathrm{d} \tau_{\nu} = \int \kappa_{\nu} S_{\nu} \mathrm{e}^{-\tau_{\nu}} \, \mathrm{d}s , $$

where $I_{\nu}$ is the specific intensity, $\tau_{\nu}$ is the optical depth, and $\kappa_{\nu}$ is the monochromatic extinction coefficient per unit path length. In the Rayleigh–Jeans limit, the equation for specific intensity can be simplified:

4 $$ I_{\nu} = \frac{2\nu^{2}k_{\mathrm{B}}T_{\mathrm{B}}}{c^{2}} . $$

Here $T_{\mathrm{B}}$ is the brightness temperature, *i.e.* the temperature a black body would have if it were to emit with the same intensity, $I_{\nu}$. Through simple comparison between Equations 2, 3, and 4, an expression for the observable brightness temperature in terms of the kinetic temperature (usually taken as the electron temperature), the local extinction coefficient, and the optical depth can be derived:

5 $$ T_{\mathrm{B}} = \int \kappa_{\nu} T \mathrm{e}^{-\tau_{\nu}} \,\mathrm{d}s . $$

Here $\mathrm{d}s$ describes an interval along a path in the LOS. Using the known temperature distribution of the model and by calculating the position- and wavelength-dependent extinction coefficient across the thread, the observable brightness temperature is calculated. In the case where $T$ is constant across the LOS, Equation 5 simplifies to

6 $$ T_{\mathrm{B}} = T\bigl(1 - \mathrm{e}^{-\tau_{\nu}}\bigr). $$

If $\tau \ll 1$ is true for an isothermal plasma, $T_{\mathrm{B}}$ becomes proportional to $T^{-\frac{1}{2}}$.

#### Calculating the Extinction Coefficient

The largest contributions to extinction in mm/sub-mm wavelengths come from free-free extinction due to inverse thermal bremsstrahlung from ionised hydrogen and helium, and to a lesser degree, from $\mathrm{H}^{-}$ extinction. Inverse thermal bremsstrahlung describes the case where an electron in the Coulomb field of an ion becomes energised through the extinction of a photon from the radiation field. In cgs units the extinction coefficient describing inverse thermal bremsstrahlung, including the stimulated emission term, is

7 $$ \kappa^{\mathrm{ff}}_{\mathrm{ion}} \approx 9.78 \times 10^{-3} \frac{n_{\mathrm{e}}}{\nu^{2}T^{\frac{3}{2}}} \sum_{i} Z_{i}^{2} n_{i} \times \bigl(17.9 + \ln T^{\frac{3}{2}} - \ln{\nu}\bigr) , $$

where $T$ is the temperature, $\nu$ is the frequency, and $n_{\mathrm{e}}$ is the electron density, $i$ represents each ion species considered, *e.g.* hydrogen or helium ions, and $n_{i}$ and $Z_{i}$ are the ion density and ion charge, respectively (Wedemeyer *et al.*, 2016). Equation 7 is evaluated from the semi-classical inverse thermal bremsstrahlung extinction in the absence of a magnetic field, given in Dulk (1985).

Continuous $\mathrm{H}^{-}$ extinction occurs from two sources, photo-detachment and free-free transitions, described in Equations 8 and 9, respectively:

8 $$\begin{aligned} & h\nu + \mathrm{H}^{-} \rightarrow \mathrm{H} + \mathrm{e}^{-} , \end{aligned}$$

9 $$\begin{aligned} & h\nu + \mathrm{e}^{-} + \mathrm{H} \rightarrow \mathrm{H} + \mathrm{e}^{-*} , \end{aligned}$$

where $\mathrm{e}^{-*}$ symbolises an energised electron. The analytical expressions describing the extinction coefficients representing these two mechanisms are given in John (1988). The total extinction coefficient from $\mathrm{H}^{-}$ extinction is considerably lower than that for inverse thermal bremsstrahlung, but at high temperatures and low wavelengths, its significance does increase. Other extinction mechanisms that have been considered include Thomson and Rayleigh scattering.

### Geometry and Integration Method

**Figure 2:**
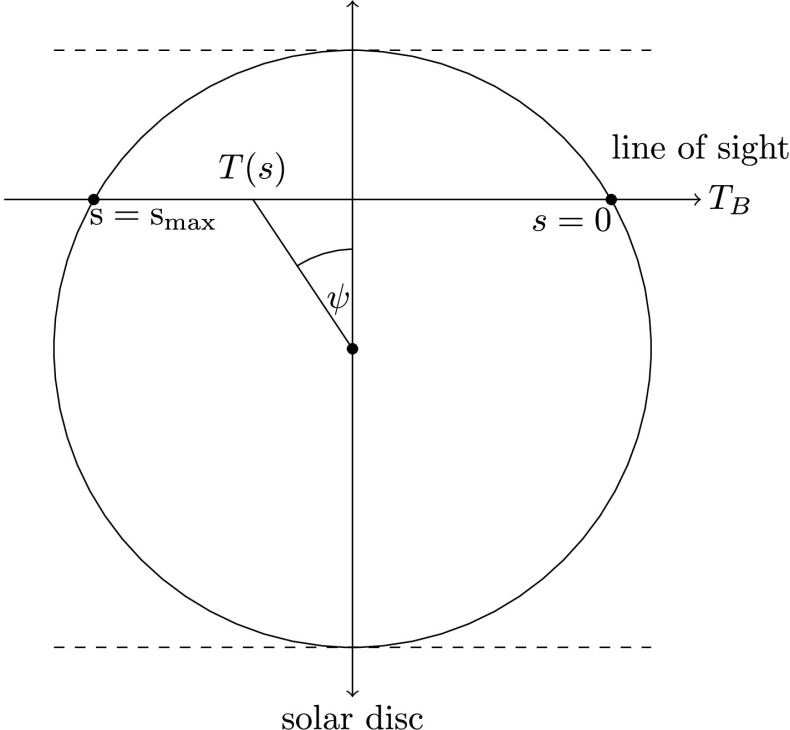
Schematic diagram showing the integration direction along the LOS. Here $s=0$ and $s=s_{\mathrm{max}}$ correspond to the start and the end of the path of light, respectively. The *dashed lines* correspond to the edges of the FOV.

Whilst C2D2E can consider any range of LOS directions or prominence inclinations, we only present results from off-limb threads here. The thread is thus orientated horizontally with respect to the solar surface, whilst the LOS of the “observer” crosses the cylindrical axis perpendicularly. The altitude of all model prominences in this study is 10 000 km. A vertical field of view (FOV) is defined such that the centre of the FOV corresponds to the cylindrical axis of the thread. For each position in the FOV the maximum length for a horizontal light path through the thread is defined. Through interpolation the local temperature and extinction coefficient are determined at every point on the path, and are then integrated in the manner described in Equation 5. The optical depth, $\tau_{\nu}$, is defined such that it is zero at the edge of the cylinder closest to the observer and maximum at the opposite end of the path. The geometry of this set-up is visualised in Figure 2.

The grid of radial and azimuthal positions defined for each thread quantity, *e.g.* electron temperature or extinction coefficient, is finite, and hence taking values at any given position along a path will require interpolation. The azimuthal grid has constant steps and is symmetric with respect to the $\psi = 0$ plane, *i.e.* it can be reduced to the range [$0,\pi$]. To interpolate across the azimuthal grid, a Fourier method is used (Gouttebroze, 2005):

10 $$ F(\psi) = \sum_{j=1}^{N_{\psi}} a_{j} \cos \bigl[(j - 1)\psi\bigr] , $$

where $N_{\psi}$ is the total number of positions in the azimuthal grid and $a_{j}$ is defined by

11 $$ a_{j} = \sum_{k=1}^{N_{\psi}} B_{jk} F_{k} . $$

The matrix, $B$, is defined solely by the azimuthal grid. Fourier-series interpolation has the advantages of smoothness and periodicity (Gouttebroze, 2005).

## Computed Brightness Temperatures

### Isothermal-isobaric Fine Structures

**Figure 3:**
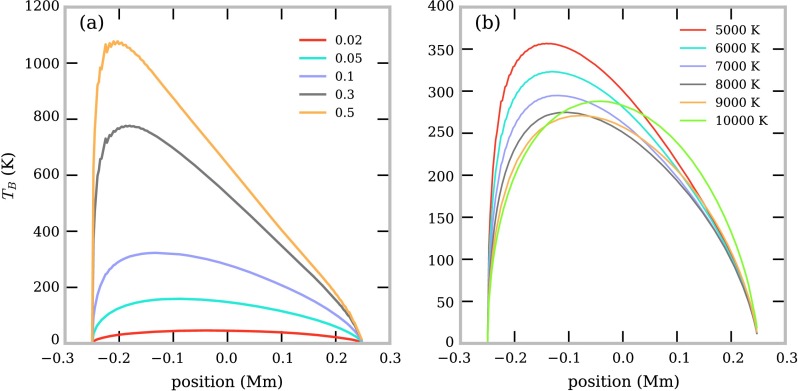
Computed brightness temperature across the FOV for ALMA Band 6, $\lambda = 1.3~\mbox{mm}$. Panel (**a**) shows the effect of increasing gas pressure ($\mbox{dyn}\,\mbox{cm}^{-2}$) on models with a temperature of 6000 K. Panel (**b**) shows the effect of increasing temperature (K) on models with a gas pressure of $0.1~\mbox{dyn}\,\mbox{cm}^{-2}$.

In Figure 3 we show the computed brightness temperature of 1.3 mm emission (ALMA band 6) across the FOV for a set of isothermal-isobaric fine-structure models. The FOV is orientated such that the position axis increases with increasing distance from the solar surface. Figure 3a shows the brightness temperature across the FOV for models with differing isobaric pressures. From the equation of state, low pressures reduce the overall density of the prominence, resulting in a lower brightness temperature. The low-density plasma allows ionising incident radiation to penetrate further through the thread, creating a symmetrical brightness temperature distribution. When we consider increasingly high pressures, the density will increase and thus too the brightness temperature. High-density threads prevent incident radiation penetrating the entire thread. This causes a relative increase of ionisation towards the thread’s lower boundary, which receives more radiation from the solar disc than the upper boundary. This in turn increases inverse-thermal bremsstrahlung absorption in this area. The higher extinction coefficient leads to higher brightness temperatures, creating an asymmetric distribution.

Figure 3b shows the brightness temperature across the FOV for models with different isothermal temperatures. At low temperatures, an increase in the kinetic temperature causes a decrease in the brightness temperatures across the FOV. This will be partly due to the decreased density, through the ideal gas law, and partly due to the relation of the inverse-thermal bremsstrahlung extinction with temperature (Equation 7). At high temperatures the increase in temperature leads to further ionisation of the neutral material, increasing inverse-thermal bremsstrahlung opacity, and thus the brightness temperature. When the material is ionised due to an overall increase in temperature, the ionising incident radiation has a less significant effect, creating a symmetrical brightness temperature distribution across the FOV.

**Figure 4:**
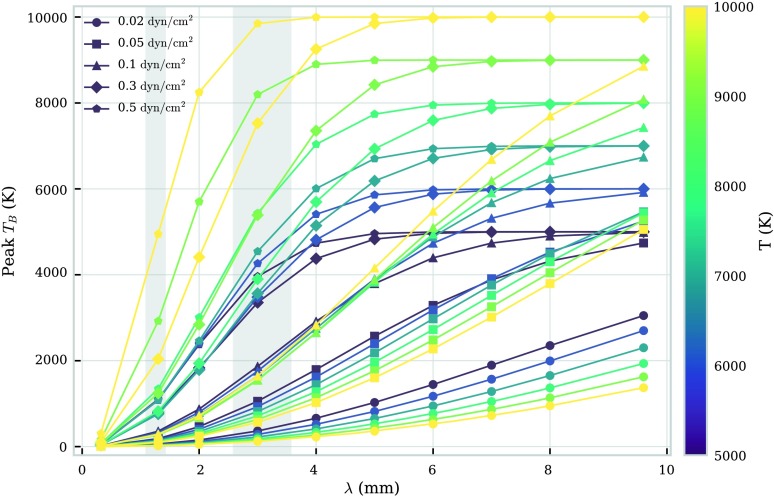
Relationship between peak brightness temperature and wavelength for a set of isothermal-isobaric fine-structure models. Each *colour* corresponds to an isothermal temperature (K), as described in the *colour bar to the right*. Each *symbol* corresponds to an isobaric pressure ($\mbox{dyn}\,\mbox{cm}^{-2}$), as described in the legend. The *two grey-shaded areas* depict ALMA observing Bands 6 and 3.

We show in Figure 4 the wavelength, temperature, and pressure dependence of the peak brightness temperature in the FOV. The peak brightness temperature will also be dependent on the radius, *i.e.* the length of the LOS, and the altitude of the prominence fine structure. Here we consider fixed values for both these quantities, whilst observationally, these values could be fairly easily constrained. From Figure 4 it can be seen that the peak brightness temperature increases with wavelength, until the point in which it reaches the temperature of the plasma. This should occur when the optical thickness of the observing wavelength reaches or exceeds unity.

The brightness temperature generally increases with wavelength due to the enhanced extinction from inverse-thermal bremsstrahlung (Equation 7). The point at which the peak brightness temperature reaches saturation with the kinetic temperature is defined by the radiation extinction coefficient, *i.e.* the higher the extinction coefficient, the lower the wavelength required to reach saturation with the kinetic temperature. The extinction coefficient is not purely wavelength dependent, but also depends on electron and ion density and temperature: the higher the pressure, the higher the density, which leads to more extinction and thus higher brightness temperatures. Increasing temperature in high-pressure models leads to more ionisation and thus higher brightness temperatures. At low pressures, the optical thickness is much lower than 1, making the brightness temperature proportional to $T^{-\frac{1}{2}}$. Increasing the temperature can also decrease the overall density more than it increases the ionisation, causing a decrease in extinction and brightness temperature.

The observed optically thick mm continuum emission is most representative of the plasma near the position where the optical depth reaches unity. For a fully isothermal thread, this measurement is thus an accurate representation of the kinetic temperature over the entire thread. Hence multiple optically thick wavelength observations will reproduce the same brightness temperature measurement, seen as the saturation features in Figure 4.

**Figure 5:**
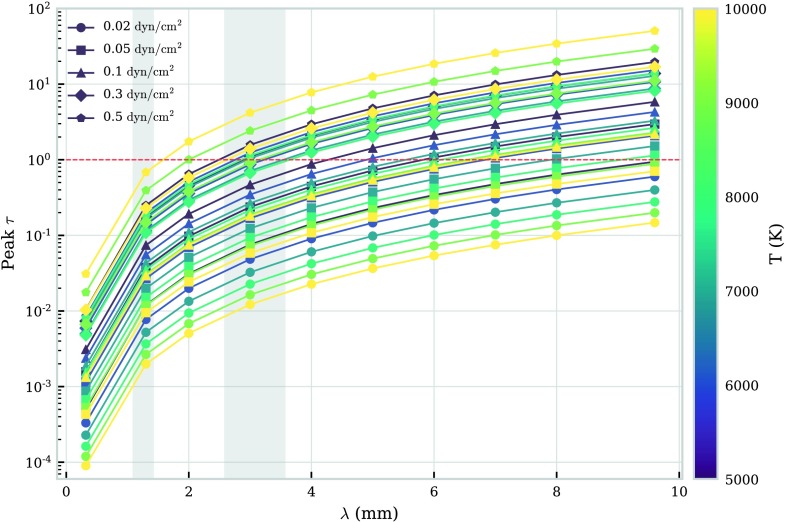
Relationship between peak optical thickness and wavelength for a set of isothermal-isobaric fine-structure models. Each *colour* corresponds to an isothermal temperature (K), as described in the *colour bar to the right*. Each *symbol* corresponds to an isobaric pressure ($\mbox{dyn}\,\mbox{cm}^{-2}$), as described in the legend. The *red-dashed line* shows the transition between optically thick and optically thin emission. The *two grey-shaded areas* depict ALMA observing Bands 6 and 3.

Owing to their limited spatial extent, individual observed fine-structure threads will naturally tend towards being optically thin in all except for the highest wavelengths and extinction. In isobaric-isothermal models with a fine structure of the scale of these threads, the peak optical thickness of Band 6 radiation fails to reach $\tau = 1$ for all models, whilst the optical thickness of Band 3 radiation exceeds $\tau = 1$ for models at pressures of 0.3 or $0.5~\mbox{dyn}\,\mbox{cm}^{-2}$ (Table 1). The relationship between wavelength and peak optical thickness for this set of isothermal-isobaric fine-structure models is shown in Figure 5. Increasing the size of the thread will increase the optical thickness at the observed wavelength.

Each individual isobaric-isothermal model produces a distinct peak brightness temperature versus wavelength curve. If geometrical variables such as altitude or LOS width can be constrained, a brightness temperature observation of an isobaric-isothermal fine-structure thread at a known wavelength could be used in conjunction with our set of models to set constraints on the pressure and temperature of the structure in consideration. When multiple observations in different wavelength bands are available, the constraints on the isobaric-isothermal model should improve greatly.

### Non-Isothermal Large-Scale Structures

**Figure 6:**
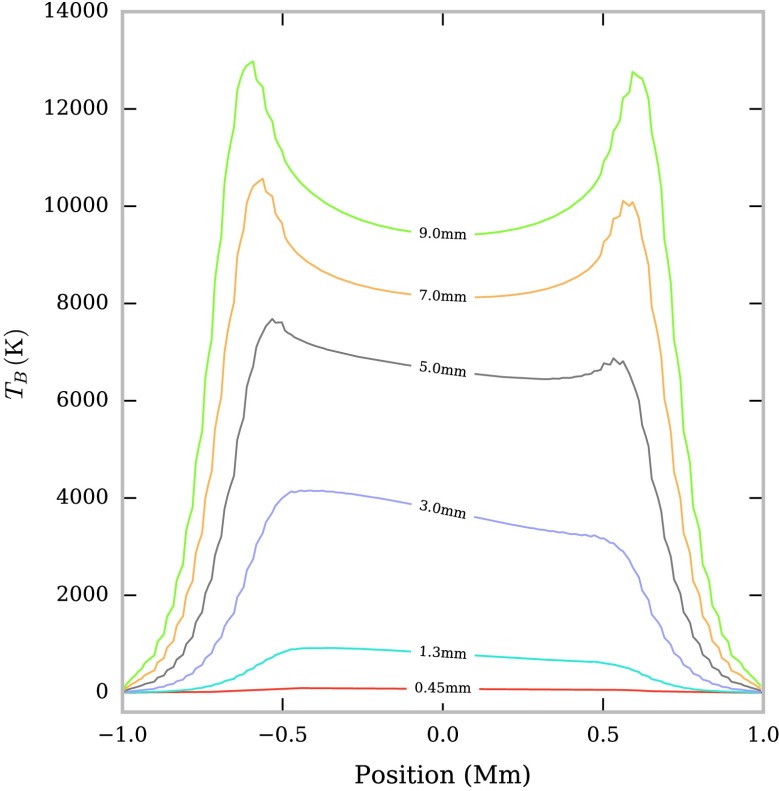
Variation in brightness temperatures across the FOV in non-isothermal large-scale prominence models. The isobaric pressure is $0.1~\mbox{dyn}\,\mbox{cm}^{-2}$ and the FOV is orientated vertically in the solar atmosphere, with the positive x-axis directed radially away from the Sun.

**Figure 7:**
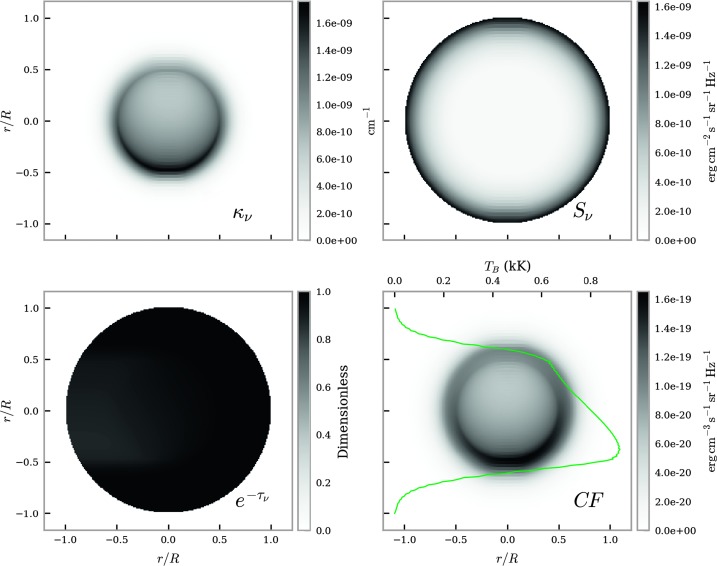
Brightness temperature FOV for non-isothermal large-scale prominences with a gas pressure of $0.1~\mbox{dyn}\,\mbox{cm}^{-2}$ at $\lambda = 1.3~\mbox{mm}$. The *top left* panel shows a map of the extinction coefficient, the *top right* panel shows the source function (Planck function), and the *bottom left* panel shows the optical thickness attenuation term. The source function here is described by the Planck function. The resulting contribution function map is plotted in the *bottom right* panel. Integrating over each horizontal LOS results in the brightness temperature (K) curve, *solid green line*.

**Figure 8:**
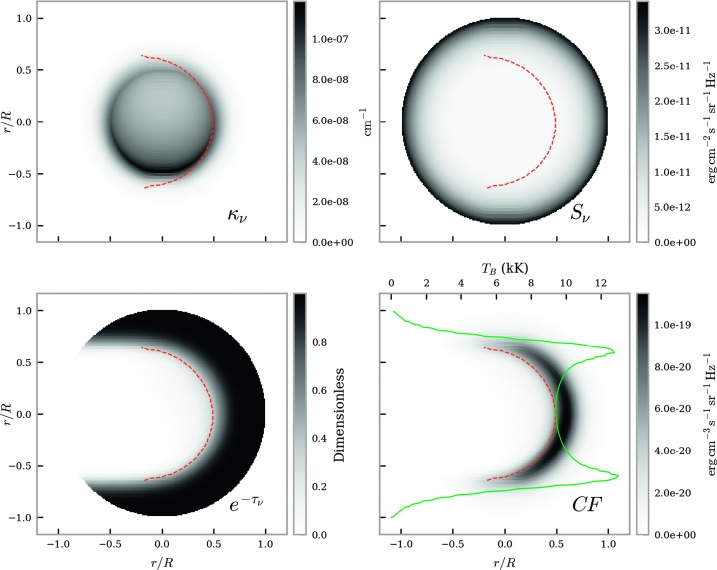
Brightness temperature FOV for non-isothermal large-scale prominences with gas pressure of $0.1~\mbox{dyn}\,\mbox{cm}^{-2}$ at $\lambda = 9~\mbox{mm}$. The *top left* panel shows a map of the extinction coefficient, the *top right* panel shows the source function, and the *bottom left* panel shows the optical thickness attenuation term. The source function here is described by the Planck function. The resulting contribution function map is plotted in the *bottom right* panel. Integrating over each horizontal LOS results in the brightness temperature (K) curve, *solid green line*. The *dashed red line* shows the $\tau = 1$ line.

In Figure 6 we show the variation in brightness temperature across the FOV for a large-scale prominence structure with a radially increasing temperature and a pressure of $0.1~\mbox{dyn}\,\mbox{cm}^{-2}$ (Table 2) at several mm/sub-mm wavelengths. It is immediately clear that there are two regimes that can describe the brightness temperature variation. The emission at 0.45, 1.3, and 3.0 mm is optically thin ($\tau < 1$) in this model, and thus displays a smooth, asymmetric variation across the FOV. Emission at 5.0, 7.0, and 9.0 mm is optically thick ($\tau \geq 1 $) and shows a nearly symmetric dual-peaked variation. The formation of these two regimes is better understood through considering the contribution function and its constituent parts (see Figures 7 and 8).

The formation plots show how the extinction coefficient, source function, and optical thickness attenuation terms combine across the thread to produce a map of the contribution function, which is shown in the bottom right panel of each figure. In this study we define the contribution function to be the direct product of the extinction coefficient, source function, and the exponential optical thickness attenuation term. Figure 7 shows the optically thin case where the attenuation, $\mathrm{e}^{-\tau_{\nu}}$, is close to 1 and nearly uniform across all LOSs in this cross-section. Photons at mm wavelengths thus travel through the thread mostly unperturbed, allowing the plasma at the far side of the LOS to contribute almost equally to the material near the surface closest to the observer. The prominence plasma at UV wavelengths is non-transparent, however, thus leading to an increase in ionising radiation incident on the lower side of the thread. This leads to higher ionisation and therefore a higher contribution function at the side of the thread that is closer to the solar disc. Integrating the contribution function along each horizontal path in the FOV results in the brightness temperature curve shown in the bottom right figure. The temperature variation is azimuthally symmetrical, hence so too is the source function (Equation 2).

Figure 8 is an example of a predominantly optically thick case, where within the central part of the thread the attenuation term has a large effect. The red-dashed line represents the $\tau = 1$ line, *i.e.* the point at which the thread becomes optically thick. The high attenuation within the central region leads to a crescent-shaped contribution function map, around the $\tau = 1$ line. The core and far side of the thread are thus under-represented in the integration over the LOS. Each of the two peaks in the brightness temperature variation corresponds to the extremal heights for which the thread is optically thick. This is due to a longer LOS intersecting through more high-temperature PCTR material. Beyond the peaks the plasma is once again optically thin, and the brightness temperature drops off quickly.

The incident radiation ionising the optically thick plasma leads to an increase in extinction coefficient, but also to an increase in attenuation from the $\mathrm{e}^{-\tau_{\nu}}$ term. This produces an almost symmetrical brightness temperature variation.

#### Thermal Diagnostic

It is difficult to determine a temperature distribution from brightness temperature measurements of optically thin plasma. The resultant brightness temperature will be an integration over potentially large temperature variations, hence losing any discernible structure. Conversely, optically thick emission is representative of a specific formation region, *i.e.* the Eddington–Barbier approximation states $T_{\mathrm{B}}(0) \approx T(\tau = 1)$.

To investigate how a brightness temperature measurement relates to the prominence plasma in the formation layer, we define an effective formation layer as the parts of the prominence where the contribution is equal to or greater than 70% of the maximum contribution function for each LOS. The effective formation temperature ($\langle T_{\mathrm{e}} \rangle_{\mathrm{fl}}$) is then found by taking the contribution function weighted mean of the temperature distribution across the effective formation layer.

**Figure 9:**
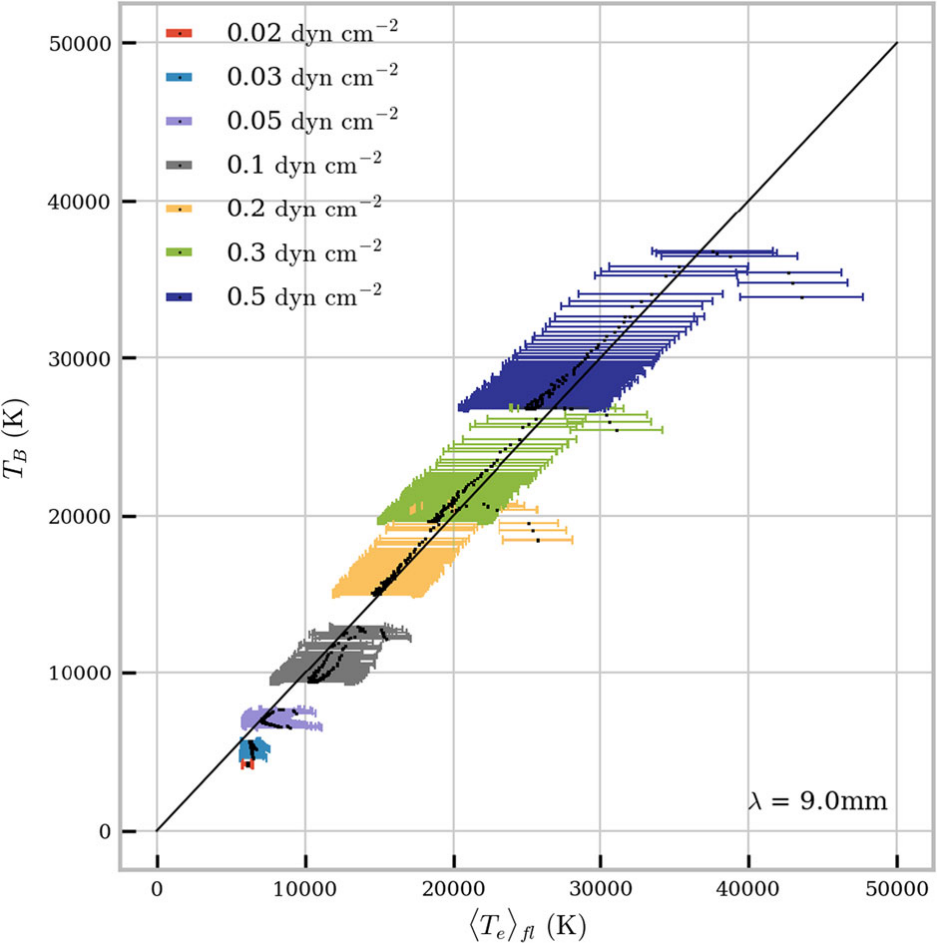
Relationship between brightness temperature and average kinetic temperature over the formation layer in the LOS. The formation layer is defined to be the region with 0.7 times the maximum contribution function or greater for each LOS in which the plasma is optically thick. The error bars show the standard error in the mean for the mean kinetic temperature. Each *colour* corresponds to a different pressure, as defined in the legend.

**Figure 10:**
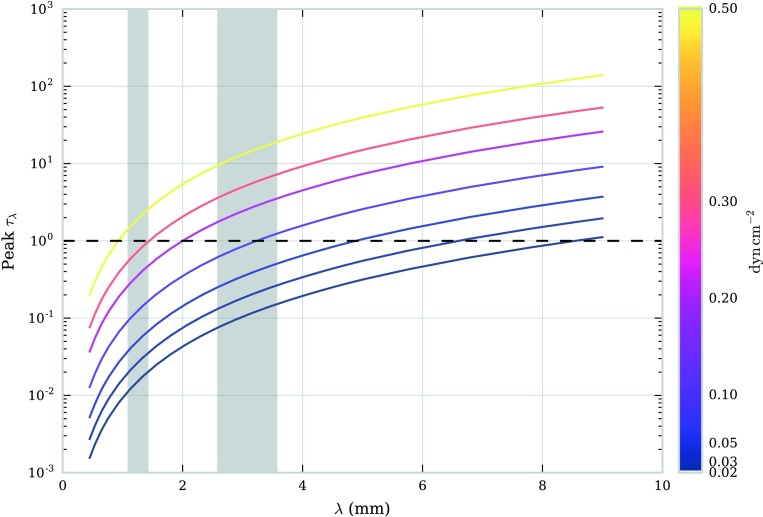
Relationship between peak optical thickness and wavelength for a set of non-isothermal large-scale structures at various pressures. The *dashed line* represents the transition between optically thin and thick plasma. The two *grey-shaded areas* cover ALMA Bands 3 and 6.

Figure 9 shows the relationship between computed brightness temperature and the mean temperature of the effective formation layer for $\lambda = 9.0~\mbox{mm}$ across a range of isobaric pressures. Each point on the graph represents an optically thick LOS. Optically thin LOSs are ignored as their contribution functions are very broad across the LOS, giving poor temperature diagnostics. At higher pressures, more of the thread is optically thick, and hence more LOS points are shown on the graph. For the majority of LOSs, the brightness temperature scales linearly with the mean temperature of the formation layer, with only some deviation at high temperatures in each model. At low pressures, the effect of lower boundary ionisation from incident radiation can again be seen through the splitting of the trend into two separate lines. Observations of brightness temperatures at optically thick wavelengths, such as $\lambda = 9.0~\mbox{mm}$, are thus fairly good indicators of the mean electron temperature of specific areas of the prominence. With high-resolution observations of multiple optically thick wavelength bands, it should be possible to build up an understanding of the temperature distribution within the prominence structure, as each wavelength band should be formed at a different formation layer. In Cycles 4 and 5 of ALMA, however, the only two bands available to solar physicists are Bands 3 and 6. These bands are significantly less optically thick than the radiation at $\lambda = 9.0~\mbox{mm}$, with $\tau$ only exceeding unity at the centre of the thread for models with high pressures. The relationship between wavelength and peak optical thickness is shown in Figure 10 for the non-isothermal models described in Table 2. The two grey-shaded areas represent ALMA Bands 3 and 6. As expected, the peak optical thickness (*i.e.* the maximum optical thickness found in each model as the LOS is varied) increases with wavelength and with pressure. Figure 10 shows that a structure of a similar size to what is modelled here (radius ${\approx}\,1000~\mbox{km}$) observed with ALMA in both Bands 3 and 6 can only be expected to be mutually optically thick at high pressures, *i.e.* greater than $0.5~\mbox{dyn}\,\mbox{cm}^{-2}$.

## Plasma Diagnostics

This section investigates the potential use of a brightness temperature ratio as a diagnostic for the optical thickness of the observed radiation and the emission measure of the emitting plasma. Here we follow a similar approach to that of Bastian, Ewell, and Zirin (1993). In the fourth observational cycle of ALMA, the two wavelength bands available to solar physicists are Band 6 (1.3 mm) and Band 3 (3.0 mm). The brightness temperature ratio, $R$, is defined as

12 $$ R = \frac{T_{\mathrm{B},1.3}}{T_{\mathrm{B},3.0}} , $$

where $T_{\mathrm{B}}$ is the brightness temperature, and the subscripts 1.3 and 3.0 denote the wavelengths at 1.3 mm and 3.0 mm, respectively. These subscripts are used throughout this section.

When a constant temperature can be assumed across the prominence, Equation 12 can be expressed in terms of the optical thicknesses of the two measurable wavelengths,

13 $$ R \approx \frac{T(1-\mathrm{e}^{-\tau_{1.3})}}{T(1-\mathrm{e}^{-\tau_{3.0}})} = \frac{1-\mathrm{e}^{-\tau_{1.3}}}{1-\mathrm{e}^{-\tau_{3.0}}} . $$

In this case, the brightness temperature ratio is related to the two optical thicknesses only. The optical thickness, at a given wavelength $i$, can then be approximated as

14 $$ \tau_{i} \approx \langle \kappa_{i} \rangle L, $$

where $\kappa_{i}$ is the wavelength-dependent continuum extinction coefficient and $L$ is the length of the LOS. Using this assumption, the optical thicknesses of the two observable wavelengths are related as follows:

15 $$ \tau_{1.3} \approx \frac{\langle \kappa_{1.3} \rangle }{ \langle \kappa_{3.0} \rangle}\tau_{3.0} = K \tau_{3.0} , $$

where $K$ has been defined as the dimensionless opacity ratio.

As described in Section 2, the largest source of opacity in the mm/sub-mm continuum is inverse thermal bremsstrahlung (Equation 7). Because of its dominance, it is hence reasonable to estimate the opacity ratio whilst only considering contribution from inverse thermal bremsstrahlung. In this case, $K$ is defined as

16 $$ K = \frac{\nu_{3.0}^{2} (17.9 + \ln (T^{3/2}) - \ln (\nu_{1.3}))}{\nu_{1.3}^{2} (17.9 + \ln (T^{3/2}) - \ln (\nu_{3.0}))} . $$

The opacity ratio is thus dependent only on the known observational frequencies and an isothermal temperature for the LOS.

**Figure 11:**
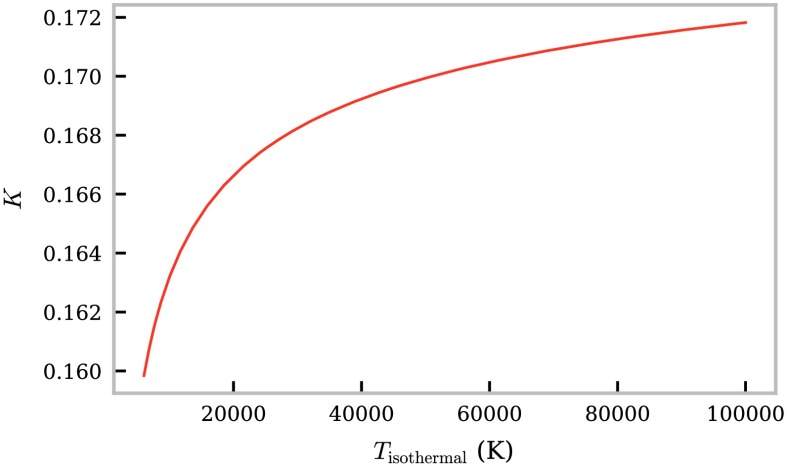
Variation in opacity ratio for ALMA wavelengths $\lambda_{1} = 1.3~\mbox{mm}$ and $\lambda_{2} = 3.0~\mbox{mm}$ with temperature (Equation 16).

To understand how the opacity ratio $K$ may vary with temperature in a prominence, it was calculated according to Equation 16 using wavelengths 1.3 mm and 3.0 mm for a range of temperatures (Figure 11) from low core temperatures of around 5000 K to extreme PCTR temperatures of $10^{5}~\mbox{K}$. From Figure 11, it is clear that $K$ varies only slightly across this temperature range, and for any expected prominence temperature, $K \ll 1$ is an acceptable assumption. When the electron temperature of the prominence is known or can be suitably assumed, a bound on the magnitude of the opacity ratio can be set.

### Estimating Optical Thickness

With a value for the opacity ratio, the optical thickness of either wavelength can be estimated by substituting Equation 15 into Equation 12. The resulting equation can be solved analytically by expanding the exponential terms only up to second order. This leads to

17 $$ \tau_{3.0} = \frac{2(K - R)}{K^{2} - R} . $$

Whilst this solution is satisfactory for high temperatures and low optical thicknesses, it was found to underestimate the optical thickness as the latter increased. To improve the estimate for higher optical thickness cases, a numerical solution must be found instead. This was done by finding the roots of the function

18 $$ f(\tau_{3.0}) = \sum_{n=1}^{N} \frac{(-1)^{n} (K^{n} - R)}{n!} \tau_{3.0}^{n-1}, $$

using the Newton–Raphson method. Here $N$ is the order to which the exponential terms are expanded. For models with the highest pressure and highest optical thickness, this method reached a constant solution for all $N \geq 18$.

We tested this method for optical thickness estimation using the same set of isothermal-isobaric fine-structure models as discussed in Section 3.1. The orientation of each prominence was as described in Section 2.3. We obtained brightness temperatures at both 1.3 mm and 3.0 mm, which were then used to calculate the ratio $R$ for all points in the FOV.

**Figure 12:**
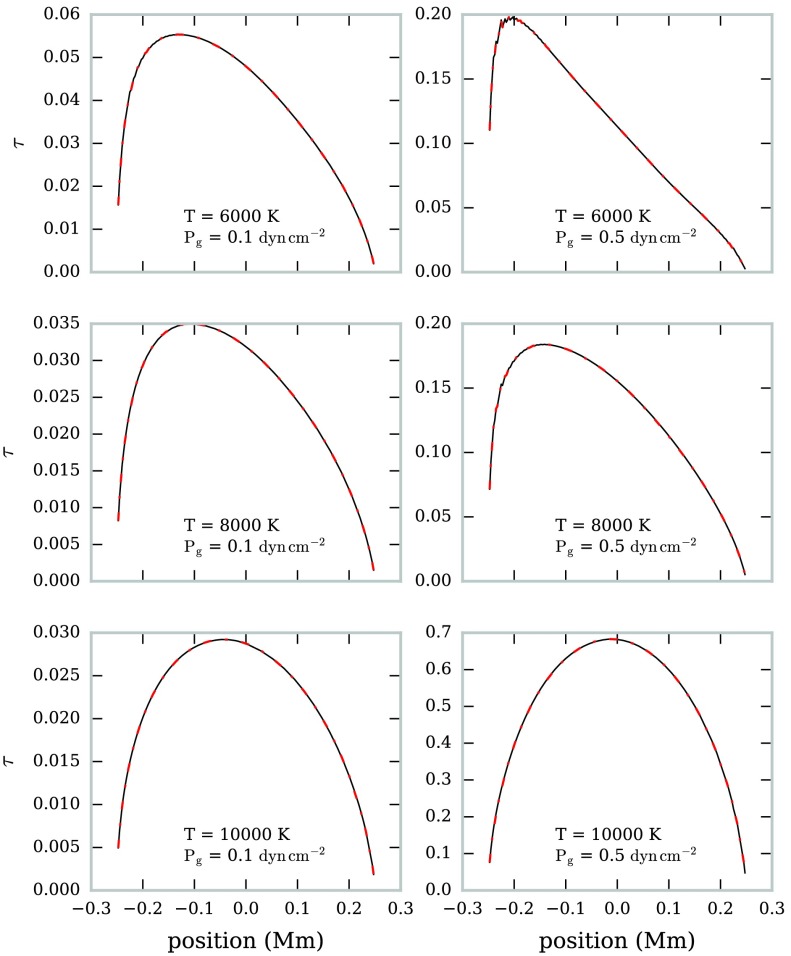
Variation in estimated (*solid black line*) and computed (*dot-dashed red line*) optical thickness with the FOV for six isothermal prominence models at $\lambda=1.3~\mbox{mm}$. The FOV is orientated vertically in the solar atmosphere, with the positive *x*-axis directed radially away from the Sun.

In Figure 12 the estimated variation in LOS optical thicknesses of the 1.3 mm radiation across the FOV is shown for a sub-set of the isothermal-isobaric models. The opacity ratio $K$ was calculated using Equation 16 and the known isothermal temperature for each model. We find that the optical thickness estimate matches the computed optical thicknesses well across a wide range of isothermal temperatures. The estimate is similarly accurate for $\lambda = 3.0~\mbox{mm}$.

It should be noted that our computed brightness temperatures are idealised and noiseless and that an attempt to use this method with real brightness temperature measurements would have an associated uncertainty. This uncertainty would likely have a significant effect when both observation wavelengths are highly optically thin, *i.e.* $\tau \ll 1$. Both brightness temperatures will be low and will hence present a low signal-to-noise ratio. Equation 13 can be simplified to $R \approx K(T)$ here.

### Estimating the Emission Measure

When the optical thickness at a given wavelength can be estimated sufficiently well, it is only a small step further to being able to estimate the average emission measure for a given LOS. Again, we assume that in the solar mm/sub-mm domain, opacity is greatly dominated by free-free inverse thermal bremsstrahlung. Substituting Equation 7 into Equation 14, the mean emission measure can be written as

19 $$ \langle \mathrm{EM} \rangle = \frac{\tau_{\nu} \nu^{2} T^{3/2}}{9.78 \times 10^{-3}(17.9 + \ln (T^{3/2}) - \ln (\nu))}, $$

where $\nu$ is the frequency of the observation, and $\mathrm{EM}$ is defined as

20 $$ \mathrm{EM} = n_{\mathrm{e}} \sum_{j} Z_{j} n_{j} L . $$

Here $n_{\mathrm{e}}$ is the electron density, with $Z_{j}$ and $n_{j}$ being the charge and density of ion species $j$, respectively.

**Figure 13:**
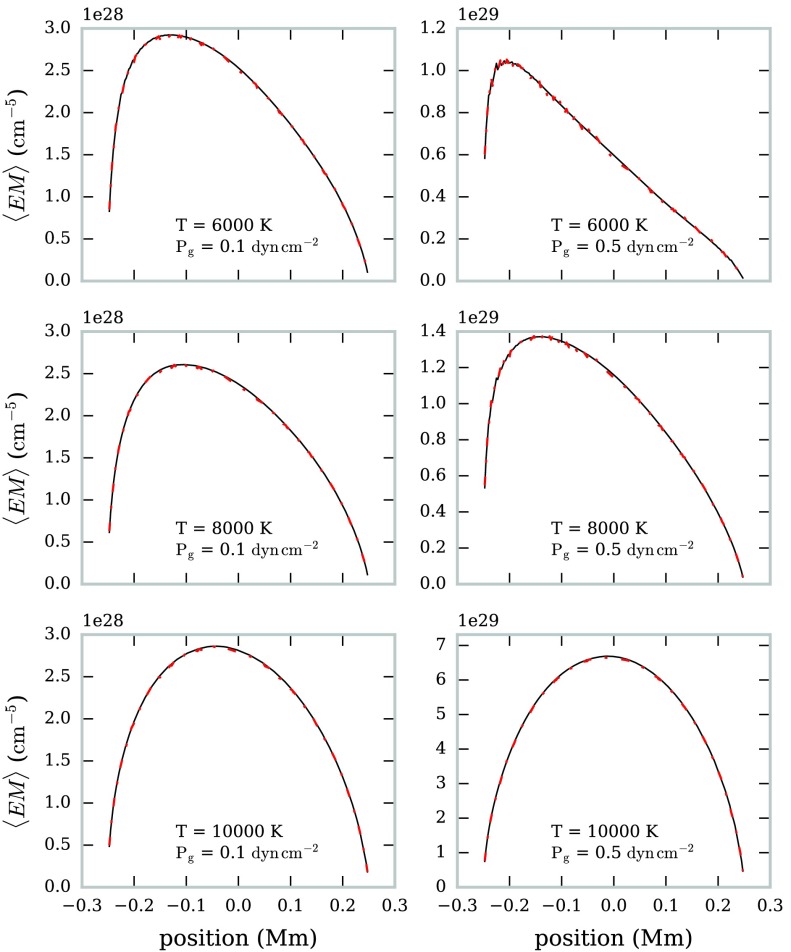
Variation in estimated (*solid black line*) and computed (*dot-dashed red line*) mean emission measure with the FOV for six isothermal prominence models. The FOV is orientated vertically in the solar atmosphere, with the positive *x*-axis directed radially away from the Sun.

We used the optical thickness values calculated in Section 4.1 to estimate the mean emission measure with this method, as seen in Figure 13. The estimated value is very close to the calculated value, with only a very slight underestimation for low isothermal temperature models. Both 1.3 mm and 3.0 mm produce the same estimate for the emission measure value.

It has been shown that the optical thickness and the emission measure can be well estimated for isothermal prominence models with known temperatures. However, a real prominence will likely have a significantly more complex temperature distribution. The next section discusses the effectiveness of these estimation methods on non-isothermal prominences with radially increasing temperature structures.

### Non-isothermal Case

**Figure 14:**
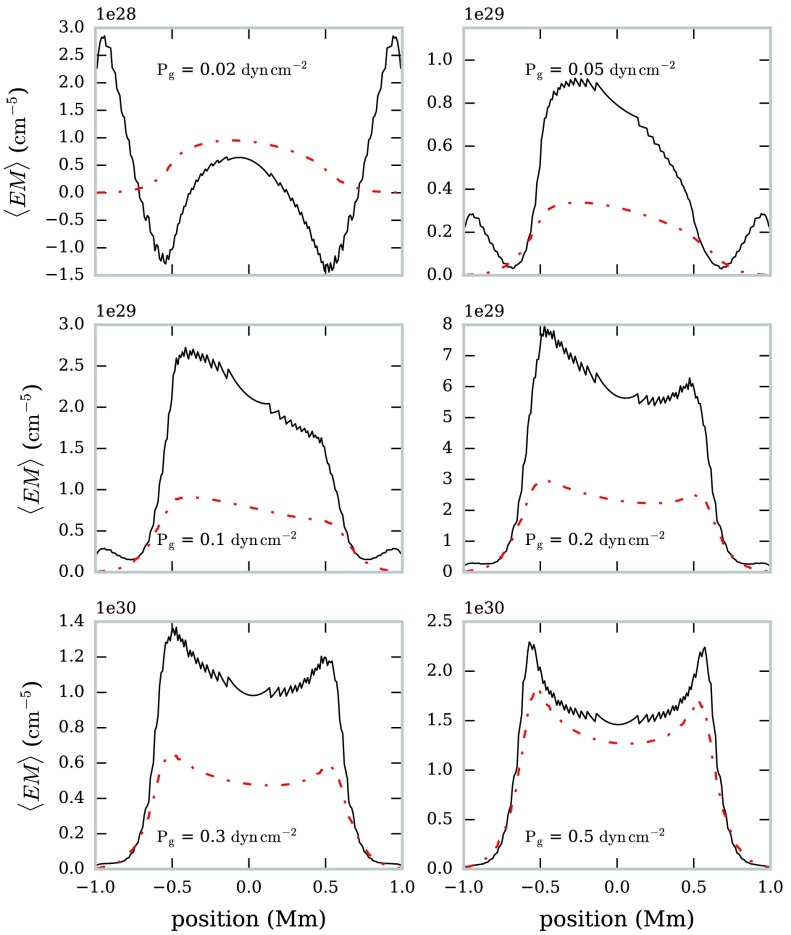
Variation in estimated (*solid black line*) and computed (*dot-dashed red line*) mean emission measure with the FOV for six non-isothermal large-scale prominence models. The FOV is orientated vertically in the solar atmosphere, with the positive *x*-axis directed radially away from the Sun.

The optical thickness and emission measure were estimated for a set of prominence models with a temperature variation increasing radially from an isothermal core (see Table 2 and Section 3.2). The brightness temperature measurements at 1.3 mm and 3.0 mm from each model were used to estimate the respective optical thicknesses and the mean emission measure, using the method described above. The average temperature for each LOS across the FOV was calculated and used as separate input temperature approximations. The average emission measure estimated for a selection of non-isothermal large-scale prominence structures estimated using this method is shown in Figure 14. The estimated values often overestimate (by up to a factor ${\approx}\, 3$) the true value of the average emission measure for each model.

For optically thin radiation, the resultant brightness temperature is a result of the integral of the contribution function across the whole LOS. However, because each LOS is non-isothermal and because of the temperature dependence of the mm emission contribution function, different layers will present different overall contributions to the output brightness temperature. Hence the output brightness temperature may not be representative of the average temperature of the LOS. In the top left panel of Figure 14, the plasma is sparse enough for the estimation to produce a negative value for the optical thickness and thus the emission measure. This is obviously unphysical and is caused by the brightness temperature ratio exceeding the opacity ratio (Equation 17), as the value for the opacity ratio is created from an unrepresentative temperature estimate for the sparse material.

In the optically thick case, the emission is representative of a small region within the LOS, and two wavelength observations will likely be representative of differing layers, and thus estimations using an average temperature for the LOS are difficult.

## Discussions and Conclusion

In this study we have modelled brightness temperatures of solar prominences in the wavelength range of ALMA. We considered a 2D cylindrical structure filled with hydrogen and helium, where the ionisation and level populations have been calculated under non-LTE conditions. Two sets of prominence models were used: isobaric isothermal small-scale structures, and large-scale structures with radially increasing temperature distributions. In both cases, ALMA is expected to be able to provide strong thermal diagnostic capabilities, provided that the interpretation of the observations is supported by the use of non-LTE simulation results.

The results of this study clearly show that whilst ALMA will present an important opportunity to improve our understanding of solar prominences, the interpretation of the observed brightness temperatures may be less straightforward than the often-used phrase “linear thermometer” might suggest. There will be several decisions observers will have to make whilst choosing how to analyse a prominence observation with ALMA. In this article we outlined two contrasting methods for approaching the analysis of prominence mm/sub-mm observations: either assuming a purely isothermal structure, or accepting the requirement for a varying temperature distribution.

The adopted approach requires knowledge of how the prominence as a whole appears when viewed with ALMA, and how its appearance is altered with observation band and interferometer array configuration. The visualisation of how whole prominences may look when observed with ALMA has been simulated by Heinzel *et al.* (2015a) and Gunár *et al.* (2016). The former used a method to convert a calibrated H$\alpha$ intensity map into an estimated brightness temperature observation. Owing to the nature of H$\alpha$ emission, the output brightness temperature map would primarily reflect the cool prominence core material. The spatial resolution of the simulated brightness temperature map is also limited by the resolution of the H$\alpha$ observation. The latter study uses a 3D whole prominence fine structure model based on dips in a model magnetic field to calculate the hydrogen free-free extinction coefficient and thus the brightness temperature for a wavelength and LOS. Both visualisation methods suggest that mm/sub-mm radiation could be observed from prominence fine structures with the resolution of ALMA. If ALMA is indeed capable of resolving individual fine-structure threads separately from the prominence as a whole, such observations could be analysed by adopting an isothermal-isobaric assumption.

In Section 3.1 we presented the results for brightness temperature calculations for a grid of isobaric-isothermal fine-structure prominence threads. For ALMA’s observational cycles 4 and 5, solar observations will have wavelength Bands 3 and 6 available. For individual fine-structure threads, these wavelengths are likely to be optically thin, unless high pressures are present. As the radiation is unlikely to be optically thick, direct measurement of the kinetic temperature from a saturated brightness temperature is unlikely. However, non-LTE isobaric-isothermal radiative transfer models, such as we have presented in this article, could be used alongside optically thin measurements to constrain isobaric pressure and isothermal temperature parameters. Multiple wavelength observations will help to constrain the models further, although cycles 4 or 5 will not offer simultaneous Band 3 and Band 6 observations according to the current status. Measurements from different channels within each band may help to improve constraints on the model by better sampling the brightness temperature versus wavelength curve, but ideally, a wider spread of wavelengths would work better.

If the isothermal-isobaric assumption were to be expanded to structures larger than the fine-structure threads discussed in this study, the optical thickness of Band 3 and perhaps Band 6 radiation may exceed $\tau = 1$, allowing direct measurement of the kinetic temperature. If the large prominence structure being observed is perceivably formed of a number of fine-structure threads, a multi-thread solution could instead be considered (Gunár *et al.*, 2008; Labrosse and Rodger, 2016). A multi-thread solution such as this may increase the optical thickness past unity, allowing direct temperature measurements to be obtained.

In Section 4 we showed that for an isothermal prominence thread the ratio of brightness temperature measurements at two wavelengths can be a useful plasma diagnostic for optical thickness and average emission measure along the LOS. This diagnostic requires the knowledge of the kinetic temperature of the observed plasma. Plasma estimations using only highly optically thin measurements may be difficult as the brightness temperatures associated with the observations would be low.

When analysing prominence observations of large-scale structures or numerous collections of fine structures, a blanket isothermal assumption will likely be invalid. To understand a varying temperature distribution such as is expected in the PCTR would require accurate positional sampling of the local kinetic temperatures throughout the structure. Optically thin emission will likely be of little use here as the resulting brightness temperature will describe contributions from across the entire LOS, presenting at best an average kinetic temperature over what could potentially be large temperature gradients. If optically thick observation is possible, the resultant brightness temperature is most representative of a localised region within the LOS around the position where $\tau = 1$. Different wavelength ALMA bands, sub-bands, and channels within sub-bands will be representative of different regions due to the sensitivity of the contribution functions to temperature. With observations across the prominence, optically thick radiation may be used to infer the temperature structure of the prominence. This will be affected by fluctuations in local density and ionisation fraction, however. Multiple observations at different optically thick wavelengths could potentially yield much-desired information on the internal temperature of solar prominences. However, from the models presented in this study with radii of 1000 km (Table 2), Bands 3 and 6 only produce peak optical thicknesses greater than unity for models with pressures greater than 0.1 and $0.5~\mbox{dyn}\,\mbox{cm}^{-2}$, respectively (Figure 10). Multiple wavelength observation within the individual channels of either band may be possible, but their close wavelengths may cause them to represent locations of similar kinetic temperature. Future ALMA cycles will provide simultaneous and co-spatial observations with more band options available to solar observers. This will allow a better understanding of the detailed temperature structure within prominences, as longer wavelength observations will more consistently correspond to optically thick radiation for larger sections of material.
